# Rapid Improvement in Cerebral Palsy Following Treatment With Perispinal Etanercept: A Case Report

**DOI:** 10.7759/cureus.114937

**Published:** 2026-08-21

**Authors:** Danielle Ucci, Samantha Asseraf, Genavieve Prieto, Alanna Tucker, Edward Tobinick

**Affiliations:** 1 Neurology, Institute of Neurological Recovery, Boca Raton, USA

**Keywords:** central pain, cerebral palsy, intraventricular hemorrhage, microglia, neuroinflammation, painful spasticity, perispinal etanercept, tnf

## Abstract

Etanercept, a selective inhibitor of the cytokine tumor necrosis factor (TNF), delivered by perispinal administration, has been used clinically for 15 years to treat chronic, intractable, post-stroke and post-brain injury neurological dysfunction. We report the case of a 19-year-old patient with cerebral palsy (CP) who experienced rapid and sustained neurological improvement after treatment with perispinal etanercept (PSE). The patient was born at 25 weeks gestation following a twin pregnancy complicated by abruptio placentae and premature onset of labor. The initial head ultrasound, performed four weeks after birth, demonstrated grade four intraventricular hemorrhage (IVH) with acquired ventriculomegaly and the patient was subsequently diagnosed with CP, hydrocephalus, and retinopathy of prematurity. She presented at age 19 to our clinic with chronic right hemiparesis, bilateral upper limb fine motor dysfunction, painful spasticity and multiple, additional chronic neurological symptoms. The patient received an initial 25mg dose of PSE, followed by doses at weeks three, 24 and 49. The patient reported reduced painful spasticity and improved sensation within 30 minutes of each dose. Clinically meaningful improvements that persisted over the entire one-year period of observation were noted in painful spasticity, sensation, gait, mobility, balance, coordination, fine motor function, strength, fatigue, and headaches; some of the improvements were progressive. PSE was well tolerated. Further study of PSE for selected patients with CP in properly designed, randomized, controlled trials is warranted.

## Introduction

Cerebral palsy (CP) is primarily characterized by impairments in movement, posture, and motor function resulting from a non-progressive injury, abnormality, or disruption of the developing brain. The clinical manifestations of CP may evolve over time and may be accompanied by impairments in sensation, cognition, communication, and other neurological functions [1].

Motor impairment is the defining clinical feature of CP and may include spasticity, weakness, impaired coordination, balance and gait abnormalities, and fatigue. Because these manifestations can affect multiple functional domains, standardized neurological and physical performance testing is important for documenting baseline impairment and objectively evaluating clinical change.

Rapid alternating movement (RAM) testing is a neurological assessment of cerebellar function, coordination, and motor control in the upper and lower extremities [2]. An impaired ability to perform alternating movements rapidly, smoothly, and rhythmically is known as dysdiadochokinesia, a finding also commonly observed following stroke [2]. RAM assessments used in the present case included finger-to-nose testing, repetitive opening and closing of the fist, forearm pronation and supination, and toe-tapping.

Handgrip strength testing, commonly performed using a hydraulic dynamometer, provides a quantitative measure of force and can assist in evaluating focal weakness and overall muscle strength [3]. The Five-Times-Sit-to-Stand Test provides an additional standardized measure of lower-extremity strength, postural control, balance, and functional mobility and has demonstrated high reliability for evaluating lower-extremity performance [4].

The brain changes associated with CP may occur before, during, or shortly after birth, or within the first two years of life while the brain is still developing [5]. Clinical presentations include spastic CP, comprising spastic hemiplegia, diplegia, and quadriplegia, as well as dyskinetic, ataxic, and mixed forms [5]. CP affects approximately 1.5 to three children per 1,000 live births and is diagnosed primarily through clinical assessment and physical examination [6]. Prematurity and low birth weight are among the most common predisposing factors, although CP has also been associated with hypoxic-ischemic encephalopathy, maternal infection, and multiple gestation [6]. In preterm infants who subsequently develop spastic CP, intracerebral hemorrhage (ICH) and periventricular leukomalacia are among the most frequently identified underlying pathological findings. Despite the prevalence of CP and the substantial disability associated with its manifestations, effective treatments for chronic neurological dysfunction remain limited.

Intraventricular hemorrhage (IVH) is a major complication of preterm birth and an important contributor to the subsequent development of CP [7]. Progression from hemorrhage confined to the ventricular system to hemorrhage extending into the brain parenchyma reflects a more extensive neurological injury, with greater potential to disrupt neurodevelopment and initiate secondary inflammatory processes. The neurodevelopmental consequences of IVH are substantial and correlate with its severity. Grade 3 IVH has been associated with CP in approximately 74% of affected infants, while grade 4 IVH carries an even greater risk, with approximately 97% of affected patients developing CP [7].

Within the CNS, tumor necrosis factor (TNF) is a signaling molecule produced by glial cells that participates in the regulation of neuronal communication and network activity [8]. Activated microglia produce TNF, while excess TNF may, in turn, stimulate further microglial activation, creating a self-reinforcing inflammatory cycle [9]. A 2023 study described this process as a TNF-driven autocrine feed-forward loop which permits neuroinflammation to persist long after the initiating injury [9]. The rapid clinical responses to etanercept reported in patients with chronic stroke and traumatic brain injury (TBI), together with evidence that persistent microglial activation contributes to neuronal dysfunction through TNF-related mechanisms, suggest that excess TNF may be more than a biomarker of inflammation and may actively exacerbate neurological dysfunction [8].

The neurological effects of IVH may also extend beyond the immediate consequences of hemorrhage. Increased levels of TNF have been reported in both patients with CP and individuals with IVH, suggesting that neuroinflammation may contribute to CP pathophysiology [5]. IVH and ICH can initiate an inflammatory response within the cerebrospinal fluid (CSF), characterized by increased concentrations of inflammatory cytokines, including TNF [10]. This cascade may not be confined to the acute injury period. Persistent neuroinflammation may contribute to the development and maintenance of neurological deficits and to the long-term clinical manifestations of CP, similar to its proposed role in chronic neurological dysfunction after stroke [8].

Evidence specifically linking TNF and inflammatory signaling to CP continues to emerge. A 2022 study concluded that elevated blood TNF levels were associated with an increased risk of CP [11]. A 2022 review identified inflammation as an important component of multiple perinatal brain injuries associated with CP, including neonatal stroke and prematurity, and described an association between elevated pro-inflammatory mediators and CP diagnosis, particularly among individuals born prematurely [12]. Persistent immune dysregulation may contribute to long-term symptom burden by increasing glial cell hyper-responsiveness and exacerbating pain, cognitive impairment, and neurological deficits [12], continuing to influence brain development long after the initial insult through ongoing cellular and molecular cascades [13].

Under this model, persistent neuroinflammation may represent a modifiable contributor to chronic symptoms rather than merely a residual marker of a remote neurological insult. This concept provides a biological rationale for considering perispinal etanercept (PSE) treatment years or even decades after the initiating neurological injury, as suggested in literature published in 2015 [14].

Excess TNF can disrupt synaptic mechanisms, impair neuronal function, and contribute centrally to the pathogenesis of multiple neuroinflammatory disorders [8]. Excess TNF’s postulated participation in the pathogenesis of secondary reversible brain injury therefore represents a potential therapeutic target in certain neuroinflammatory disorders, including stroke, TBI, and CP. Etanercept is a biologic TNF inhibitor that binds TNF and reduces its biological activity, and etanercept also reduces microglial activation.

Perispinal administration followed by Trendelenburg positioning has been proposed as a method of enhancing delivery of etanercept to the CNS using the cerebrospinal venous system (CSVS) as a unique vascular conduit [15]. PSE has been used in clinical practice for more than 15 years for patients with chronic neurological dysfunction following stroke and TBI. Clinical evidence has documented neurological improvement in post-stroke and post-TBI patients treated with PSE years or even decades after the initiating event [8]. A 2023 review suggested that reduction of excess cerebral TNF may interrupt persistent, TNF-maintained microglial activation and thereby improve neurological function [9].

TNF-mediated neuroinflammation may contribute to central post-stroke pain (CPSP) and may also play a mechanistic role in the chronic pain and neurological dysfunction experienced by some patients with CP [10]. Pain is highly prevalent in the pediatric CP population, affecting as many as 85% of children, with prevalence increasing among older children and females [16]. Chronic pain can substantially affect mobility, participation, sleep, emotional well-being, and quality of life. The literature has also identified similarities between chronic pain in CP and CPSP, raising the possibility that treatments capable of reducing CPSP may have relevance for selected patients with CP [13].

We present a case report that examines the response of a patient with CP to PSE, with particular attention to pain, spasticity, coordination, muscle strength, balance, mobility, and other neurological functions. Standardized clinical assessments were incorporated to provide objective measures of functional performance and to supplement the patient’s reported clinical response.

## Case presentation

The patient, a 19-year-old left-handed female, was born at 25 weeks gestation following a twin pregnancy complicated by abruptio placentae and premature onset of labor. Initial head ultrasound, performed four weeks after birth, showed grade four IVH with acquired ventriculomegaly. She was diagnosed with CP, hydrocephalus, and retinopathy of immaturity.

At age 18, the patient and her family contacted our clinic for consultation. Before treatment, previous medical records were reviewed, and the patient underwent blood testing, which revealed a normal complete blood count and negative screening results for latent tuberculosis. The potential risks and limitations of PSE were discussed and informed consent was obtained prior to treatment.

At age 19, the patient presented to the clinic for an evaluation, which revealed asymmetric spastic quadreparesis, more pronounced on the right than the left. She also reported various degrees of the following symptoms: dysphagia, dysarthria, dysphonia, brain fog, fatigue, balance difficulties, and intermittent headaches. Sensory symptoms included numbness of the left ribcage and bilateral feet, as well as right upper and lower extremity allodynia. Constant mild-to-severe painful spasticity involved the right jaw, right shoulder, bilateral lower back, left knee, and bilateral ankles. Baseline data points were collected before the treatment using standardized measures and physical evaluation (Table 1).

**Table 1:** Evaluation Results Serial pre-treatment clinical measures across four perispinal etanercept (PSE) treatment visits. Higher values indicate improvement for repetition-based measures and grip strength; lower values indicate improvement for timed tests and Fatigue Assessment Scale scores. Abbreviations: TX = treatment/dose; TX1 = first treatment/dose; TX2 = second treatment/dose; TX3 = third treatment/dose; TX4 = fourth treatment/dose. Finger to Nose: The patient alternates contact with their nose and the examiner’s finger as quickly as possible for 10 seconds. Fist Open and Close: The patient opens and closes their hand as quickly as possible for 5 seconds. Forearm Pronation and Supination: The patient places their hand palm-down on the thigh and quickly alternates between flipping it palm-up and palm-down as quickly as possible for 5 seconds. Toe Tapping: The patient taps the ball of their foot against the floor as quickly as possible for 5 seconds. Hand Grip Strength: The patient squeezes a standard hydraulic hand dynamometer for grip strength measurements in kilograms. Five-Times-Sit-to-Stand: The patient, starting from a seated position, stands up and sits down as quickly as possible for five repetitions. Gait: The patient walks down the hallway at normal walking speed for 20 meters.

Clinical Measures	Pre TX1	Pre TX2	Pre TX3	Pre TX4
Finger to Nose (Right) (Repetitions)	2.5	4.5	4.5	5
Finger to Nose (Left) (Repetitions)	3.5	5.5	5.5	5.5
Fist Open and Close (Right) (Repetitions)	4	5	6	6.5
Fist Open and Close (Left) (Repetitions)	4	4.5	5	6.5
Forearm Pronate and Supinate (Right) (Repetitions)	3.5	4.5	4.5	4.5
Forearm Pronate and Supinate (Left) (Repetitions)	4	5	4.5	6
Toe Taps (Right) (Repetitions)	0	4	6	6
Toe Taps (Left) (Repetitions)	9.5	8.5	13	14
Hand Grip Strength (Right) (Kilograms)	5	12	8	10
Hand Grip Strength (Left) (Kilograms)	17	22	21	24
Five-Times-Sit-to-Stand (Seconds) [4]	15	13	9	Not assessed
Gait (Seconds) (20-meters)	32	25	25	Not assessed
Fatigue Assessment Scale (Score out of 50) [17]	20 / 50	23 / 50	20 / 50	12 / 50

**Figure 1:**
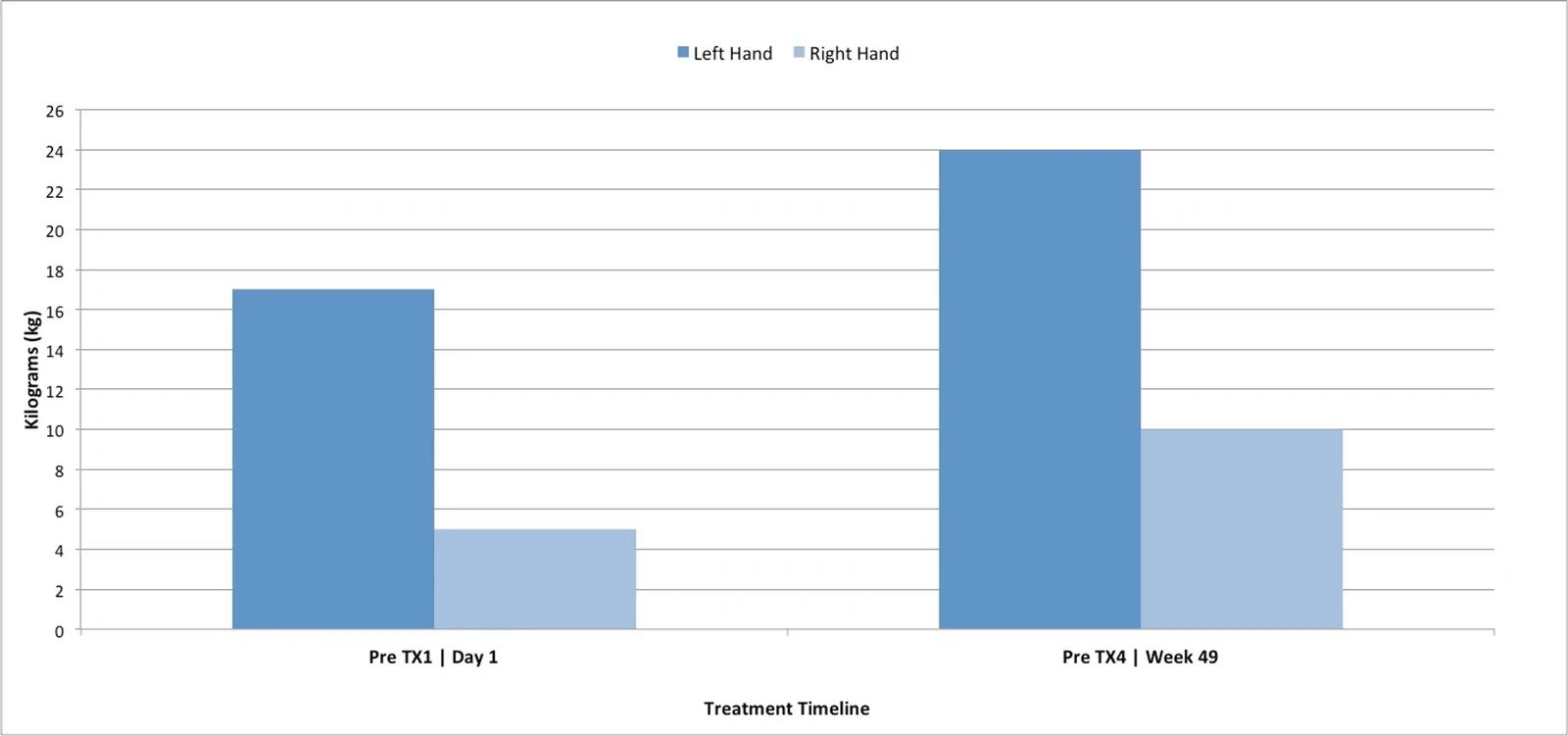
Hand Grip Strength Data Bar graph comparing right-hand and left-hand grip strength across two assessment time points: pre-treatment one (TX1) and pre-treatment four (TX4).

Following the initial clinical evaluation, the patient was treated with 25 mg of etanercept administered perispinally at the C6-C7 vertebral level and was then placed in Trendelenburg positioning for seven minutes. Within 30 minutes of treatment, the patient reported reduced painful spasticity and improved sensation on the right side. Objective testing showed increased hand grip strength from 17 kg to 22 kg on the left and from 5 kg to 6 kg on the right (Figure 1). Repeat RAM testing showed bilateral improvement across all measures. No adverse effects were noted, other than injection-site discomfort, which resolved within minutes after local ice pack application.

Three weeks after the initial PSE dose, the patient reported sustained improvements in movement, sensation, painful spasticity, speech, gait, balance, coordination, dysphagia, and stamina, as well as improvement in her intermittent headaches. Objective testing conducted before the second treatment (Pre TX2) showed that right-hand grip strength increased from a baseline of 5 kg to 12 kg, and left-hand grip strength increased from a baseline of 17 kg to 22 kg (Figure 1). Twenty-meter gait time improved from 32 seconds at baseline to 25 seconds. RAM testing improved bilaterally across all measures (Table 1). No adverse effects were reported. Following clinical evaluation, the patient was treated with a second dose of 25 mg PSE, administered in the same manner as the first. Within 30 minutes, the patient reported further reduction in painful spasticity and improved gait speed. No adverse effects were noted, other than temporary injection-site discomfort.

**Figure 2:**
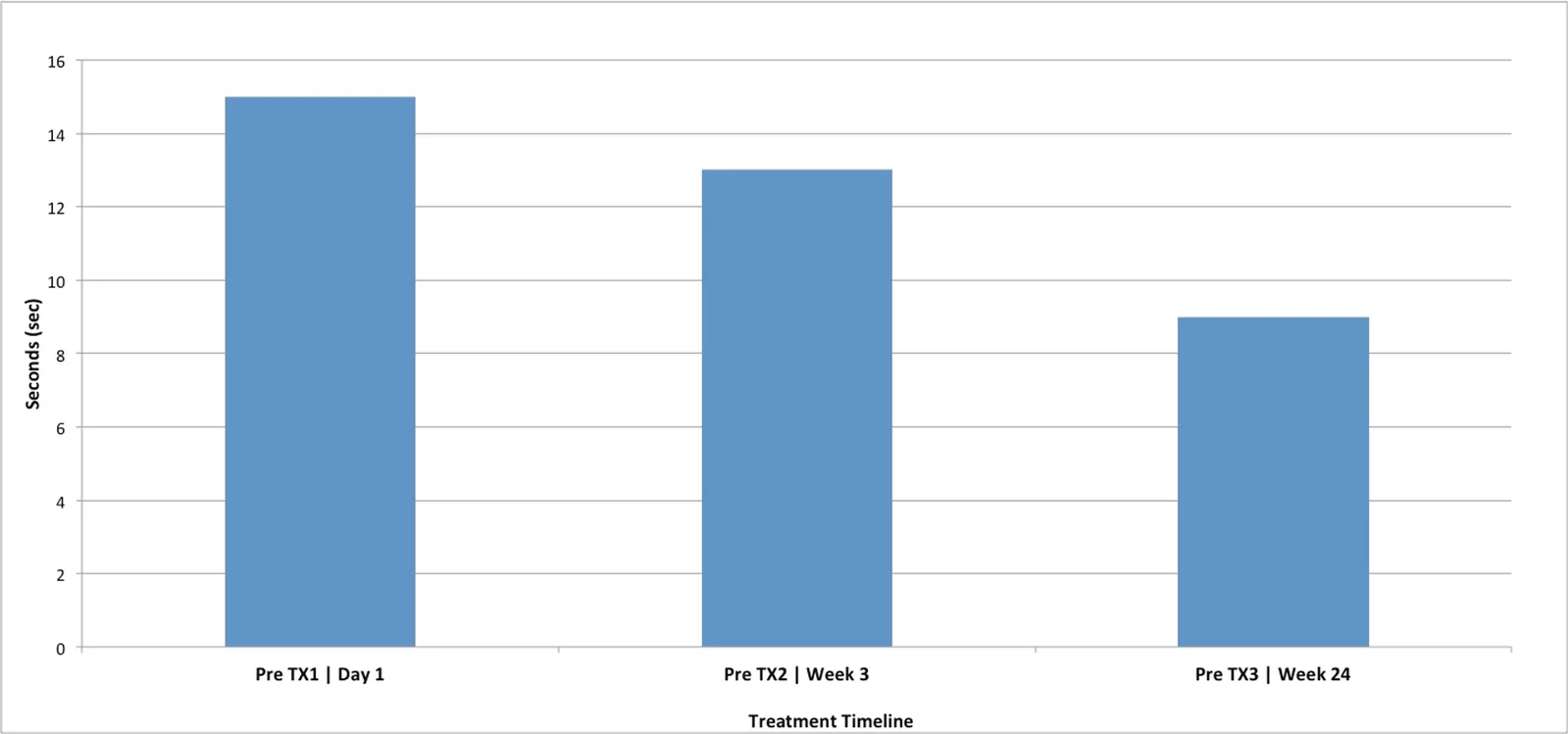
Five-Times-Sit-to-Stand Data Bar graph comparing standardized Five-Times-Sit-to-Stand test [4] across three assessment time points: pre-treatment one (TX1), pre-treatment two (TX2), and pre-treatment three (TX3).

Twenty-four weeks after the initial PSE dose, the patient reported sustained improvements in gait, cognition, and balance. Objective testing conducted before her third treatment (Pre TX3) showed improved bilateral RAM testing (Table 1) and improved Five-Times-Sit-to-Stand time (Figure 2) [4]. Following clinical evaluation, the patient was treated with a third dose of 25 mg PSE, administered in the same fashion as the first. Within 30 minutes, the patient reported reduced painful spasticity, further reduction in numbness on the right side, and improved coordination and fine motor skills bilaterally. No adverse effects were noted, other than temporary injection-site discomfort.

Forty-nine weeks after the initial PSE dose, the patient reported improvements in voice projection, which then regressed over time, and maintained improvements in fatigue and balance. Objective testing conducted before her fourth treatment (Pre TX4) showed sustained improvement in grip strength and RAM testing compared with baseline, as well as a reduced Fatigue Assessment Scale score (Table 1) [17]. Following clinical evaluation, the patient was treated with a fourth dose of 25 mg PSE, administered in the same manner as the first. Within 30 minutes, the patient reported reduced painful spasticity and improved voice projection. No adverse effects were noted, other than temporary injection-site discomfort.

Objective and patient-reported improvements were maintained at the third, 24th, and 49th week follow-up intervals after PSE treatment. These improvements involved painful spasticity, sensation, fine motor performance, grip strength, coordination, balance, gait, and fatigue.

Maintained improvements in fine motor performance, across multiple RAM tests, were observed bilaterally with scores trending upward from Pre TX1 to Pre TX4 with improvements carried over between treatments. Grip strength similarly reflected durable improvement, with right-hand strength rising and remaining above baseline across subsequent treatments. Maintained improvements in balance and coordination were observed as reflected by improved gait speed and a progressive reduction in five-times-sit-to-stand completion time from Pre TX1 to Pre TX3 (Table 1). Additionally, Fatigue Assessment Scale score decreased from 20/50 at Pre TX1 to 12/50 at Pre TX4, indicating reduced global fatigue (Table 1) [17].

## Discussion

Over the course of a year, this patient demonstrated rapid and sustained improvements across multiple clinical domains, including painful spasticity, sensation, fine motor performance, grip strength, coordination, balance, gait, and fatigue. The onset of neurological improvement, within 30 minutes of each injection, is consistent with the proposed mechanism of enhanced central delivery of etanercept after perispinal injection followed by Trendelenburg positioning and the postulated scientific rationale. Improvements in objective performance measures and patient-reported benefits across the follow-up assessments at the third, 24th, and 49th week indicate a maintained clinical benefit.

**Figure 3:**
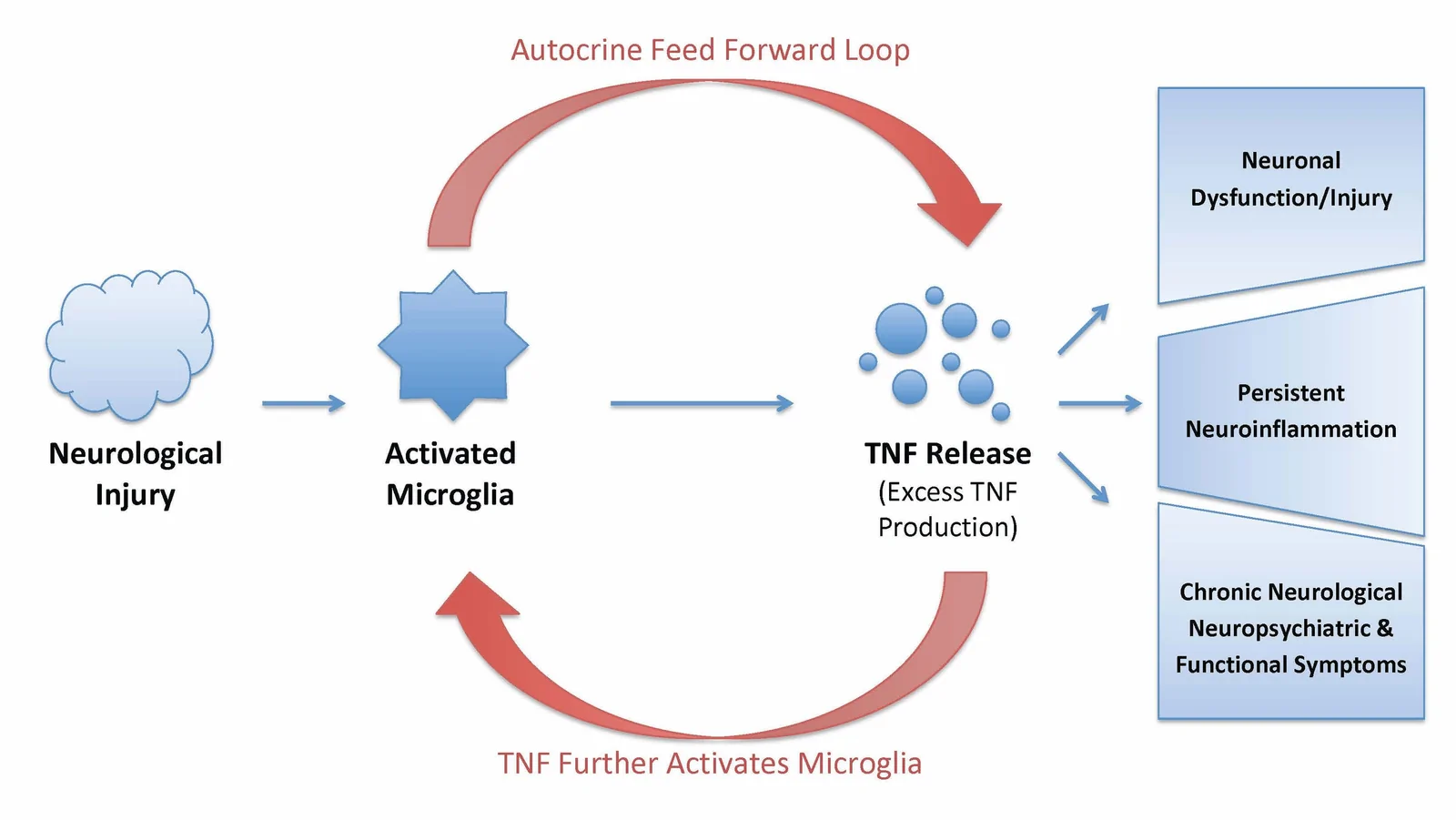
Tumor Necrosis Factor (TNF)-Driven Autocrine Feed-Forward Loop in Microglia. Diagram illustrating a proposed mechanism through which neurological injury is sustained. Elevated TNF further stimulates microglia through an autocrine feed-forward loop, promoting continued microglial activation and TNF release and potentially contributing to persistent neurological dysfunction. Figure created by the authors using Microsoft Word (Redmond, WA, USA).

These findings do not imply reversal of the original structural brain injury but raise the possibility that potentially reversible physiological processes are present, including persistent neuroinflammation and TNF-mediated synaptic dysfunction associated with the TNF-driven autocrine feed-forward loop in microglia (Figure 3) [9]. The shared features of neurological injury and persistent neuroinflammation observed in adults and in preterm infants following IVH provide support for considering TNF inhibition in selected patients with CP. Consistent with this rationale, intracerebroventricular etanercept administered after hypoxic-ischemic injury in a preterm fetal sheep model reduced white matter damage and improved myelin repair, suggesting that delayed TNF blockade may reduce neurological injury and potentially lower the risk of CP following preterm birth [9]. This model provides additional evidence that TNF-mediated inflammatory processes may influence neurological function and white matter integrity following perinatal brain injury. Preclinical models of perinatal stroke have similarly implicated disordered inflammatory processes in its pathogenesis [18].

Previous CP literature has similarly proposed that therapies targeting activated microglia, astrocytes, or inflammatory cytokines may reduce neurological injury [14]. The present case raises the possibility that these inflammatory processes may remain therapeutically relevant into young adulthood in selected patients. This is also consistent with research challenging the traditional characterizations of CP as a static injury and therapeutically unmodifiable [18].

A 2014 study found that the timing of brain injury influences the distribution of upper limb weakness, highlighting the importance of understanding underlying neuroanatomical mechanisms and neural plasticity when selecting appropriate, targeted interventions [19]. The authors suggested that when injury occurs early in development, nerve pathways from the unaffected hemisphere may remain connected to the affected limb and contribute to motor control [19]. The proposed corticospinal organization is clinically relevant to the present case, as the patient’s injury occurred during extreme prematurity and was followed by chronic right hemiparesis and bilateral fine-motor dysfunction. The observed bilateral improvements in hand grip strength and RAM performance following PSE suggest that physiological function within these developmentally reorganized motor networks may have remained modifiable through young adulthood, although corticospinal connectivity was not directly evaluated in this case. The same study further proposed that findings from adult stroke may help inform research and treatment development for postnatal neurological injury [19]. Given the improvements across multiple domains observed in this patient following PSE administration, the TNF-driven autocrine feed-forward loop involving persistent microglial activation and elevated TNF may represent one potentially modifiable neurobiological mechanism contributing to persistent neurological dysfunction. 

Conventional management of CP-related neurological dysfunction typically requires a combination of continuing rehabilitation, intramuscular injections, or surgical intervention [1,5]. These treatments can provide meaningful benefits but often target specific muscles or address particular functional limitations, rather than addressing the broader range of neurological symptoms observed in the present case.

Spasticity is often among the earliest apparent clinical manifestations of CP. Although painful spasticity is primarily nociceptive, pain in CP may also include a central component. Spasticity in CP is often treated using a combination of intramuscular injections and surgical intervention. Botulinum toxin Type A may be administered directly into affected muscles to temporarily reduce muscle tension, but may contribute to muscle atrophy over time [1,20]. When intramuscular injections are insufficient, invasive surgical intervention is used [1,20]. When evaluating CP for treatment, central pain should be considered alongside spasticity as part of the broader mosaic of symptoms that could potentially respond, based on clinical experience and the postulated underlying pathophysiology [21]. When compared with existing treatments, the changes following minimally invasive PSE in this patient were not confined to a single muscle group or to muscle tone, but they included bilateral fine motor performance, grip strength, sensation, gait, balance, coordination, fatigue, speech-related symptoms, and painful spasticity. 

The literature regarding CP and stroke also suggests that there should be overlap when approaching therapeutic interventions, as seen with shared symptoms and treatments such as botulinum toxin, muscle relaxants, and physical therapy. The shared pathogenic mechanism of neuroinflammation between stroke and CP argues for consideration of PSE as a potential treatment option for CP patients, particularly those with symptoms suggestive of continuing neuroinflammation, such as central pain, painful spasticity, fatigue and cognitive dysfunction. 

Although the CP literature generally emphasizes the greatest potential for neurological modification during early development [1,5], PSE provides an option for patients who have not achieved their desired improvement from early intervention. This potential to expand treatment options to the adult and adolescent CP populations warrants further investigation of PSE as a potential treatment for patients with CP and neurological dysfunction associated with persistent neuroinflammation.

This report has limitations. As a single-patient case report, it cannot establish causality, determine the expected magnitude or duration of the treatment effect, or quantify the contribution of placebo effects. In addition, the patient’s symptoms and objective measures were assessed in a clinical treatment setting rather than in a blinded, controlled trial. Randomized clinical trials studying clinical populations, such as those with central pain, using primary outcome measures known to be responsive to PSE, longer follow-up, appropriate controls, and physicians who have received expert training by experts in the correct methods of perispinal etanercept administration are needed to further evaluate the safety and efficacy of PSE in patients with CP.

The neurological deficits in CP, which arise early in development, often before CNS maturity, may limit the therapeutic potential of PSE in CP relative to its reported efficacy in various forms of acquired adult brain injury, such as stroke and TBI. Physical limitations which can develop after decades of spasticity, such as contractures, are unlikely to significantly improve, although central pain, if present, may potentially respond.

Clinical trials will be necessary to determine the optimal dosing schedule for patients with CP and to assess whether early intervention with PSE may alter long-term adult outcomes. Based on our clinical experience, patients with CP are likely to require ongoing, periodic maintenance treatment with PSE for optimal results; however, given the limited experience described in this single CP case, the generalizability of these findings to the broader CP population remains uncertain. Based on our experience with PSE in patients with chronic stroke, particular patient subgroups, including those with central pain or cognitive dysfunction, may be more likely to respond favorably than others.

## Conclusions

Our long-term favorable experience using PSE for chronic stroke and other forms of brain injury, and for a small number of adult patients with CP, suggests PSE may have therapeutic potential for adult patients with intractable CP-related neurological symptoms, such as central pain, spasticity, sensory symptoms, gait impairment, fatigue, balance difficulties, and fine motor dysfunction. We hypothesize that the rapid and sustained improvements observed in this case may suggest that some chronic CP-related symptoms remain modifiable in adulthood. Given the limited treatment options available for patients with CP and central pain, long-term favorable clinical experience using PSE for central post-stroke pain, a plausible scientific rationale, and a successful published peer-reviewed, double-blind, placebo-controlled, randomized clinical trial of perispinal etanercept for central post-stroke pain, warrant the initiation of PSE randomized clinical trials for adult CP patients with intractable central pain.
